# Around the EQUATOR With Clin‐STAR: Systematic Reviews of Intervention Effectiveness; Challenges and Opportunities

**DOI:** 10.1111/jgs.19498

**Published:** 2025-05-02

**Authors:** Thomas F. Crocker, Oliver Todd, Andrew Clegg

**Affiliations:** ^1^ Academic Unit for Ageing and Stroke Research Leeds Institute of Health Sciences, University of Leeds Leeds UK; ^2^ Academic Unit for Ageing and Stroke Research Bradford Institute for Health Research, Bradford Teaching Hospitals NHS Foundation Trust Bradford UK

**Keywords:** EQUATOR, geriatrics, PRISMA, reporting standards, systematic review

## Abstract

Systematic reviews offer clarity about the effectiveness of interventions based on the best available evidence. The Preferred Reporting Items for Systematic reviews and Meta‐Analyses (PRISMA) 2020 statement updated previous guidance to ensure transparency in the reporting of systematic reviews of intervention effectiveness. To adhere to the 27 items of PRISMA 2020, aging‐focused researchers must specify the choice of age‐based criteria for the review, comprehensively identify studies, select the most important outcomes that will therefore be collected, define the effect measures (e.g., odds ratio), describe investigations of the causes of heterogeneity (e.g., different settings), assess and report the risk of bias including blinding of participants and missing outcome data for results that contribute to each meta‐analysis, and discuss the implications of the findings for practice, which will often include some uncertainty. This article provides guidance on overcoming the specific challenges faced by aging‐focused researchers in transparently reporting a systematic review.

## Graphical abstract

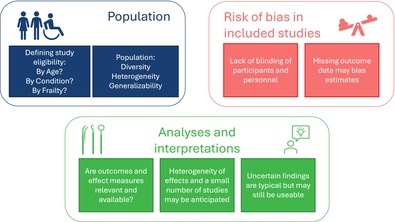

**Summary**

- Key points
  ○ For systematic reviews, the EQUATOR Network recommends the 27‐item Preferred Reporting Items for Systematic reviews and Meta‐Analyses (PRISMA) 2020 statement as the primary reporting guideline.
  ○ Specific challenges for aging‐focused researchers include the study criteria, search strategy, outcomes of interest, effect measures, investigation of heterogeneity, risk of bias, and implications for practice.
  ○ This article presents the challenges faced by aging‐focused researchers and guidance for successfully dealing with them.
- Why does this paper matter?
  ○ Systematic reviews are well established as key instruments of evidence‐based healthcare, but methodological challenges inherent to aging research mean that there are particular considerations that deserve greater guidance to ensure the best use of existing data for older people.
  ○ Transparent reporting of aging‐focused systematic reviews empowers healthcare providers to base practice on the best available evidence, improving the lives of older people.

## 1 Introduction

Systematic reviews of intervention effectiveness studies, such as randomized controlled trials, aggregate the existing evidence to provide a best estimate of the size, direction, and consistency of any effects, and an understanding of how certain that estimate is [1, 2]. To do this, they seek to identify every eligible study, evaluate eligibility against clear criteria, extract relevant data accurately, appraise the risk of bias in the extracted data, synthesize data for important outcomes, and evaluate the strength of the synthesized findings [1]. Synthesis of quantitative outcome data is typically conducted using meta‐analysis. To ensure findings are not biased by the decision making of the reviewers, methods should be prespecified and, to demonstrate this, a review can be prospectively registered on a public database such as the International prospective register of systematic reviews (PROSPERO) and the protocol published [3, 4]. Because they aim to provide a best estimate of effectiveness based on all relevant studies, systematic reviews underpin many of the statements in clinical guidelines, and therefore have a substantial influence on clinical practice [5, 6].

Systematic reviews aim to achieve transparency about the process by which they identify, synthesize, and appraise the available evidence, a task made easier with reporting guidelines [7]. The Quality of Reporting of Meta‐analyses (QUOROM) statement (1999) was developed in the face of evidence of widespread poor reporting and followed the success of the Consolidated Standards of Reporting Trials (CONSORT) guidance for randomized controlled trials [8, 9].

The Enhancing the QUAlity and Transparency Of health Research (EQUATOR) Network (https://www.equator‐network.org/) was established in 2008 to improve the reporting of health research by providing a library of reporting guidelines, supporting the development of guidelines, promoting their use, and auditing the quality of reporting [7, 10]. Shortly after this, the QUOROM statement was updated to become the Preferred Reporting Items for Systematic reviews and Meta‐Analyses (PRISMA) statement (2009) [11]. Recently, the PRISMA 2020 statement was developed as a further update based on the EQUATOR Network guidance and published (2021) across multiple journals with an accompanying checklist, and explanation and elaboration article [12, 13]. Careful consideration of PRISMA 2020 when planning a systematic review in aging research, alongside methodological guidance such as the Cochrane handbook and Joanna Briggs Institute approach, will help researchers conduct high‐quality systematic reviews and report them transparently [1, 12, 13, 14]. Many medical journals require systematic review submissions to be accompanied by a completed PRISMA 2020 checklist [15].

Systematic reviews in aging research are likely to face several consistent challenges in the application of PRISMA guidelines [16]. The objective of this review is to highlight methodological challenges that are likely to be encountered when conducting systematic reviews that are particularly relevant to research questions involving older people. We propose guidance for addressing these challenges that could be adopted by researchers conducting systematic reviews and meta‐analyses in aging research.

## 2 Systematic Reviews: Challenges and Guidance for Aging‐Focused Researchers

**FIGURE 1:**
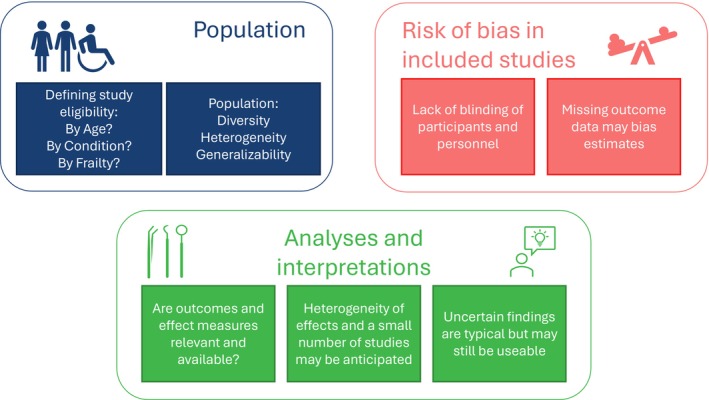
Key considerations when developing systematic reviews for older people.

PRISMA 2020 provides reporting guidelines for systematic reviews and meta‐analyses of intervention studies [12, 13]. We highlight particular domains from the 27‐item PRISMA 2020 checklist which have relevant considerations for aging‐focused researchers. These considerations are illustrated in Figure 1 and recommendations are summarized in Table 1.

**TABLE 1:** Barriers and recommendations for reviewers of aging research in following the PRISMA statement.

PRISMA section and topic	Challenges	Recommendations to overcome challenges
5. Methods: Eligibility criteria	Population often difficult to define Age‐based definitions, use of means, cultural appropriateness Frailty Lack of clear conceptual model linking precise population and intervention	Typically use a minimum average age rather than absolute cutoff [17] Consider that an age criterion may not be necessary if intervention relates itself to an age‐related condition—e.g., dementia [18, 19]. Use criteria that are specific, explicit and likely to be evaluable based on published reports [18].
7. Methods: Search strategy	Because the population can be difficult to define, it can be difficult to identify studies comprehensively	Involve medical librarians with domain‐specific expertise and awareness of the requirements of systematic reviews [20, 21].
10a. Methods: Data items	Age‐related conditions and interventions often encompass a broad range of outcomes of interest, each measured in heterogeneous ways. It is hard to define which will be included/excluded. What has previously been measured may not align with the priorities of older adults.	Involve older people and their carers alongside representatives of the study's intended audience (e.g., health professionals, commissioners) in selection and importance of outcomes [17, 22]. Consider including indicators of essential mechanisms [23]. Report examples of the measures for each outcome and a process for deciding which to include/exclude [24].
12. Methods: Effect measures	Lack of consistency in reporting and divergent interpretations of different measures, such as for falls or institutional admissions.	Consider the most widely reported metric and whether it is sufficiently meaningful/important. Use the odds ratio for binary outcomes but present absolute differences [17]. Prespecify the effect measure where possible [25].
13e. Methods: Synthesis methods	Many plausible causes of heterogeneity. Difficult to prespecify which to investigate. Often few studies contributing to analyses. Often substantial collinearity between possible sources of heterogeneity limiting interpretation. Trial results are usually presented for the study population, not stratified. Therefore, attempts to investigate heterogeneity are at risk of aggregation bias.	Prespecify the planned subgroups and explain any changes [25, 26]. Acknowledge collinearity and other limitations. Prespecify the circumstances where investigation of heterogeneity will or will not be conducted [25, 27].
18. Risk of bias in studies	Inability to blind participants and personnel in many rehabilitation interventions.	Acknowledge the risks that unblinded participants and personnel pose but consider how likely it is that this has led to sufficient performance bias to affect the results [28]. Also consider how seriously this may have affected ascertainment (i.e., biased reporting due to knowledge of what was assigned) for self‐report measures. This will depend on the measure, timing and context [29]. If there are some concerns rather than serious concerns this is not a reason to downgrade certainty in the evidence if using the GRADE approach [30].
20a. Results of syntheses (characteristics)	Missing outcome data will often be substantial for aging research studies due to follow‐up being stopped at nursing home admission, moving home, and/or death. Additionally, follow‐up may not have been well reported (which requires specific numbers for each result, reasons for missingness).	Between group differences are more important than overall missingness [31, 32]. Consider contacting study authors for further details [18]. Alternatively, consider conducting sensitivity analyses under a range of plausible assumptions [33].
23d. Discussion (implications)	Results may not be statistically significant and assessments of certainty will often not provide high certainty	Acknowledge uncertainty and the need for due caution [17, 18]. Make appropriate practice and policy recommendations accounting for uncertainty [17]. Consider clinical importance and effect size [17, 34].

### 2.1 PRISMA Methods: Eligibility Criteria

PRISMA recommends researchers specify the explicit inclusion and exclusion criteria for the review and how studies were grouped for the syntheses. The criteria are vital in operationalizing the research question and choosing all and only relevant studies.

When applying PRISMA in aging research reviews, there are a number of important considerations relating to age‐based eligibility criteria. For example, a person's chronological age is often specified as a criterion, but whether this should be an absolute cut‐off (e.g., exclude studies that include participants under 70 years of age) or an average for the population is a relevant consideration. Chronological age alone is insufficient to capture variation in the aging process among individuals [35]. Different age thresholds may be relevant in different settings, for example where age‐related conditions might be expected to occur in younger populations, often driven by the social determinants of health. For example, research studies in Australia and New Zealand may have different age cutoffs for Aboriginal, Māori and Torres Strait islanders, reflecting emergence of important health conditions at younger age in these groups [36, 37]. A person's chronological age, defined as the number of years alive, is not necessarily equivalent to their biological age, as measured, for example, by frailty, a condition characterized by loss of biological reserves, failure of physiological mechanisms, and resulting vulnerability to a range of adverse health outcomes [38]. Biological measures of age may therefore be more appropriate metrics for inclusion, but they can be measured in a variety of ways in trials and are often not reported [17].

Researchers should consider the population to which they want to generalize the evidence. They should consider the benefits and drawbacks of criteria that include a broader range of studies (more data, more heterogeneity). We would recommend using a minimum average age unless there is a clear rationale for restricting the entire population to some cutoff. For example, Crocker et al. limited a review to populations with a mean age of 65 years or over; they included 129 studies, 25 of which would have been excluded if the criterion was all participants aged 65 years or older, but almost all of these studies explicitly targeted older adults and had more than 80% of participants aged at least 65 [17]. Criteria may also be introduced to limit to studies that are expected to be more generalizable or those that explicitly include the target population. For example, if the population of interest is older people with frailty, consider excluding studies targeting a single condition. When setting their criteria, aging‐focused researchers should also consider the data that are typically reported, both now and in much older studies where these are relevant to the question. A scoping review of recent geriatric emergency medicine research reported that age criteria were usually applied but without rationale or adjustment for accelerated aging [39]. If criteria cannot be evaluated from the reports, the eligibility of many studies may be unclear, despite contacting authors [40]. These studies will be excluded from analyses even though they may have been eligible, reducing power and potentially introducing bias.

For some age‐related conditions (e.g., dementia, Parkinson's disease) it may be appropriate to ignore age as a criterion entirely, particularly if this reflects the likely organization of services [18, 19]; if concerns remain then subgroup or sensitivity analyses could be specified. In any case, it is important that the criteria are explicit and specific to enable their consistent application.

### 2.2 PRISMA Methods: Search Strategy

PRISMA requires the full search strategy for each database to be published to ensure transparency and replicability. They also recommend providing detail about the search strategy development, such as the use of validated filters and strategies adopted or adapted from previous systematic reviews, as well as peer review by trained librarians. Defining aging participants/populations is not simple (see “eligibility criteria,” below); therefore, relevant studies will have taken different approaches, and identifying such studies requires a multitude of keywords and subject headings (examples [20, 21]). Working with medical librarians with expertise in systematic reviews and aging is invaluable for developing rigorous search strategies and adapting them to an appropriate selection of databases [41].

### 2.3 PRISMA Methods: Data Items

PRISMA makes it clear that all outcomes of interest should be specified. There are often many outcome domains that are clinically relevant in aging research reviews, and many more are likely to have been reported in the included studies. Given the limitations of reviewer resources and consumer capacity, it is important to limit the domains, while remaining transparent about how decisions were taken. Specifying which are the main/critical outcomes and providing a rationale clarifies the purpose of the review.

Usually, reviews will be intended to inform clinical practice and therefore the outcomes that are most pertinent to these decisions should be prioritized. We recommend identifying and prioritizing outcomes relevant to the older person and health and social care systems by consulting the relevant stakeholders, including patients, their carers, clinicians and commissioners [22]. Consider specifying ‘composite’ outcomes (e.g., alive and in their own home/death or institutional care), or scoring systems that incorporate death (e.g., EuroQol 5 dimensions index, modified Rankin Scale) to reduce problems with missing data (see PRISMA results: results of syntheses (characteristics), below) [42, 43, 44, 45]. Core outcome sets may have been developed that help identify the most important domains to include, such as those for deprescribing in hospital and frailty [46, 47]. Consulting with stakeholders in an inclusive way remains challenging where many in the population have significant cognitive difficulties such as dementia, or communication difficulties such as aphasia following stroke, but core outcome sets have also been developed for these populations [48, 49].

On the other hand, prioritizing only distal, patient‐centered outcomes may lead to findings of no effect that do not provide direction for future research. For example, in a systematic review of interventions on reducing anticholinergic burden, a review found no clear evidence that interventions targeting anticholinergic burden in older people reduce overall anticholinergic burden score, improve cognition, impact quality of life‐related outcomes, or reduce falls [23]. However, key among these findings is that there did not appear to be a clear intervention effect on anticholinergic burden scores measured postintervention. This is of importance as it is unlikely that downstream effects on measures of cognition or other outcomes can be generated, or attributed to the intervention, in the absence of a reduction in anticholinergic burden scores. Investigating evidence of impact on mechanistic and proximal outcomes may therefore be more informative for scientific understanding and further intervention development. Presenting a logic model for complex interventions, for which effectiveness usually depends on interactions between multiple components, levels, their context, and their implementation [50, 51], is a recommended additional element to item 3: *describe the rationale for the review in the context of existing knowledge* [13]. For example, a systematic review of the effect of intergenerational activities on the mental health and well‐being of older people developed a logic model to illustrate how the effects might arise, and used this to guide their data collection and analysis [52].

It is also necessary to report precisely which measures of an outcome will be collected [25]. Quality of life is a key outcome of relevance in many aging research studies, however, outcomes measuring “quality of life” are widely conceptualized and operationalized; to limit heterogeneity and streamline data collection it may be pertinent to more tightly define such outcomes and provide examples of measures that will or will not be collected [24, 53].

### 2.4 PRISMA Methods: Effect Measures

As well as the choice of outcome measures, the effect measures (i.e., the way the difference is calculated, such as mean difference or odds ratio) should be prespecified. This is partly a matter of choosing binary, continuous, or time‐to‐event measures. Again, it is worth considering what is likely to have been reported to enable use of the available data.

We recommend aging‐focused researchers take a pragmatic approach to choosing the effect measure. This is particularly relevant for the clinical care of older people for which there is frequently a lack of evidence, study designs are heterogeneous, and they are undertaken in a multitude of different service designs and settings, such that the best attempt should be made to collate existing evidence.

Therefore, for example, while time‐to‐event data might be preferable for institutionalization, only binary data may be available. Outcomes such as hospital admissions are likely to have been reported in multiple ways (number of patients with any admissions, number of patients with 3 or more admissions, etc.) that should not be synthesized together. Given the likely heterogeneity in baseline risk in aging studies, we recommend use of the odds ratio for calculating binary effect measures due to its portability [54]. However, odds ratios are difficult to interpret and often misinterpreted as the relative risk [55, 56]; to avoid this, the estimated effect should also be presented as a risk ratio and absolute risk difference for a range of plausible baseline risks [17, 30]. Prespecification of the effect measure helps to reduce unnecessary data extraction [25].

### 2.5 PRISMA Methods: Synthesis Methods

Heterogeneity is commonly identified in systematic review syntheses and should also be investigated to see if the source can be identified, typically through subgroup analyses or meta‐regression [57, 58].

Investigation of heterogeneity often provides a dilemma for aging‐focused researchers given that there are often few such studies contributing to analyses, many plausible causes of heterogeneity, and substantial collinearity between them. Researchers should prespecify plausible factors and decide between a small number of the most scientifically cogent factors to investigate [59], or a longer list allowing many comparisons (examples [25, 26]). The longer list, although more likely to identify a source of heterogeneity, is also more likely to identify a false positive finding due to multiple testing. Either way, collinearities (measured or assumed) should be acknowledged. In developing the sources of heterogeneity to test, consideration should again be given to data availability. In particular, treatment‐covariate interactions, or raw data grouped by participant characteristics (e.g., fit/frail, male/female) are rarely provided and therefore cannot be appropriately explored [27, 60]. Reviewers of aging research should consider prespecifying when investigation of heterogeneity will occur, and possibly different approaches, depending on the number of available studies and distribution of factors among them [25, 27]. This provides transparency and enables the investigation of heterogeneity to be appropriate to the available data. The Cochrane Handbook recommends relevant considerations in the approach to heterogeneity [61].

### 2.6 PRISMA Methods: Risk of Bias

PRISMA recommends that the risk of bias be assessed for all included studies.

For reviewers of aging research, it is pertinent that for many complex interventions relevant to aging research, it is not always possible to blind both trial participants and personnel (intervention deliverers) given the nature of the intervention. While this risk needs acknowledgement, consideration should be given to whether there is any evidence of nonprotocol interventions (such as personnel choosing to deliver an experimental intervention to control participants, or control participants seeking substitute treatments due to feeling unlucky) and whether it is likely that this has led to sufficient performance bias to affect the results to a degree that is clinically meaningful [28]. Also consider how seriously this may have affected ascertainment of the outcome (i.e., biased reporting due to knowledge of what was assigned) for self‐report measures. This will depend on the measure, timing, and context (e.g., daily recording vs. 3‐monthly recall of falls [29]). If there are some concerns rather than serious concerns, this is not necessarily a reason to downgrade certainty in the evidence if using the Grading of Recommendations, Assessment, Development and Evaluation (GRADE) approach [30].

### 2.7 PRISMA Results: Results of Syntheses (Characteristics)

PRISMA recommends that for each synthesis the characteristics be briefly summarized alongside the risk of bias among contributing studies.

The external validity of trial populations is often a concern, and may limit the generalizability of findings to routine clinical practice. For example, randomized control trials in hypertension treatment often select populations who have less frailty and multimorbidity and are therefore better able to tolerate treatment [62, 63]. Both the trial explicit (reported) and implicit (not reported) exclusions are worthy of the reviewer's attention. For example, looking carefully at the number of those enrolled, as a proportion of those eligible may reveal older people were excluded on the basis of a clinical decision of the physician which is not defined [64]. In order to enroll people from hard‐to‐reach groups or population groups traditionally not included in research, including older people, particular effort needs to be made to overcome obstacles. For example, the exclusion of older people with cognitive impairment may not be explicitly listed in a trial, but is implicit if there are not provisions in the trial recruitment methods to include proxy consent on behalf of a person with variable capacity to consent, as is often the case in people living with cognitive impairment. Therefore, the missing people who were not eligible or eligible but not enrolled should be given particular attention to understand the applicability of the results to clinical practice.

For reviewers of aging research, it is also relevant to consider that a substantial proportion of older people's outcome data will often be missing as participants have died (for nonmortality/composite outcomes), or follow‐up may have stopped or contact been lost due to an adverse event or admission to a nursing home [65, 66]. Missing data due to death or nursing home admission is clearly associated with deterioration in a person's condition. Therefore, such reasons will often be associated with the value of a clinical outcome, producing a theoretical risk of bias with unknown direction, and untestable due to the missingness of the data [67, 68, 69]. Additionally, the risk of bias due to this missingness may vary widely between different outcomes for the same study (e.g., there may be more complete data for mortality than a patient‐reported outcome measure (PROM)) [70]. Therefore, it can be important to assess this risk for each study result rather than per study. The risk due to an imbalance in missingness between groups is greater than that due to overall missingness; suggestions have been made for their relative importance [31, 32]. However, this loss to follow‐up may not have been well reported (specific numbers for each group and result, including reasons for missingness). In these instances, ways forward include contacting study authors for further details [18]. Alternatively, consider conducting sensitivity analyses under a range of plausible assumptions [33].

### 2.8 PRISMA Discussion: Implications

PRISMA recommends that the review discuss the implications of the results for practice, policy, and future research.

For reviews of aging research, it is important to acknowledge that there will often not be a high degree of certainty in the findings, and that implications for practice and policy must be developed in this context. Using the GRADE approach to assessing certainty, missing outcome data, unexplained heterogeneity, differences between the trial and target populations, or meta‐analysis results that cross the no‐effect line are among the reasons that certainty in findings may be reduced [5]. It is important to acknowledge uncertainty and encourage readers to exercise due caution when interpreting the results. However, where the evidence is good enough, we recommend authors make appropriate recommendations. For example, if the best estimate of effect (the point estimate) is for clinically important benefit, a marginal lack of statistical significance that excludes harm reduces certainty, but the evidence may still be sufficient to recommend action in the absence of other problems. An illustration of this from a systematic review of community‐based complex interventions for older people was the recommendation of service models that incorporate care planning with an embedded medication review despite lack of statistical significance (odds ratio of living at home 1.22, 95% confidence interval 0.93 to 1.59) as the evidence was of moderate certainty overall; in contrast, aids and adaptations alone were not recommended (or discouraged) as evidence was very uncertain despite a favorable, statistically significant estimate [17].

Here we highlight the importance of clinical significance, but this interpretation is severely complicated by uncertainty regarding the size of effect that should be considered clinically important [71, 72]. Many scales do not have published minimal clinically important differences (MCIDs), and the many ways of deriving these can lead to widely varying results [73]. Moreover, MCIDs are defined for individuals, with varying preferences, but the evidence from meta‐analyses relates to group‐level effects, where smaller effects may be considered clinically important [72]. We recommend consideration of anchor‐based MCIDs where they are available, distribution‐based MCIDs, clinical judgment, and patient and public involvement when considering clinical importance. Given the uncertainty, one approach where multiple MCIDs have been derived for included scales is to present the estimated effects alongside these MCIDs [34]. Another approach that can help with the interpretation of the results is to qualify the size of effect (e.g., small, large), perhaps using a distribution‐based approach [17]. This allows effect sizes of uncertain importance to be labeled small or very small, avoiding either ruling out or overstating the importance of an effect.

Where uncertain or no evidence has been found for an older population, aging‐focused reviewers may consider the transferability of evidence from systematic reviews of related interventions, or other populations, for which there may be good evidence [74]. Reviewers should also identify understudied populations and recommend their pro‐active enrollment in future trials. Clinical practice is almost always based on imperfect evidence; given the available evidence we must decide whether it is better to act or not [75].

## 3 PRISMA Extensions

Fifteen extensions to the PRISMA statement are hosted on the EQUATOR network (https://www.equator‐network.org/reporting‐guidelines/prisma/), addressing particular aspects, topics, and designs of systematic reviews such as the protocol, search, equity, and network meta‐analysis [76, 77, 78]. Individual Participant Data Meta‐Analysis (IPDMA) is an alternative approach to synthesis that requires acquisition of the original trial data. IPDMA enables analyses with greater power and reduced heterogeneity, producing personalized estimates of treatment effects and overcoming many of the challenges described here [79, 80]. However, IPDMA requires considerably more resources than an equivalent standard systematic review with aggregate data, and there are multiple considerations for its reporting not covered by PRISMA 2020. The PRISMA‐IPD extension covers these considerations [81]. Additionally, systematic reviews of intervention effectiveness that do not include a meta‐analysis are common, and the methods of synthesis are often poorly reported [82]. While many aspects of PRISMA apply to these systematic reviews, the guidance on synthesis methods (item 13) and results of syntheses (item 20) are specific to meta‐analysis. The Synthesis Without Meta‐analysis (SWiM) reporting guideline expands on the existing guidance in these areas [82]. Consideration of the specific challenges faced by aging‐focused researchers in applying both of these extensions is beyond the scope of the current article but would make useful additions to the Around the EQUATOR series.

## 4 Conclusions

PRISMA 2020 is an important reporting standard and compliance with its items is often mandated by journals. For the authors of PRISMA 2020 and methodological guidance such as the Cochrane Handbook, greater guidance on how to navigate these challenges that are particularly problematic in aging research may strengthen the quality of the evidence base across clinical fields [1, 12, 13, 14]. For aging‐focused researchers, we hope that consideration of the challenges and opportunities elaborated in this article will ease this process, enabling systematic reviews that better inform geriatric clinical care.

## Author Contributions

Andrew Clegg and Thomas F. Crocker contributed to the study conception. All authors contributed to the design, drafting the work, and reviewing it critically for important intellectual content.

## Conflicts of Interest

The authors declare no conflicts of interest.
